# Psoas muscle CT radiomics-based machine learning models to predict response to infliximab in patients with Crohn’s disease

**DOI:** 10.1080/07853890.2025.2527954

**Published:** 2025-07-05

**Authors:** Zhuoyan Chen, Weimin Cai, Yuanhang He, Tianhao Mei, Yuxuan Zhang, Shiyu Li, Yiwen Hong, Yuhao Chen, Huiya Ying, Yuan Zeng, Fujun Yu

**Affiliations:** ^a^ Department of Gastroenterology, Affiliated Dongyang Hospital of Wenzhou Medical University, Dongyang, China; ^b^ Department of Gastroenterology, the First Affiliated Hospital of Wenzhou Medical University, Wenzhou, Zhejiang, China; ^c^ Department of Gastroenterology, Wenzhou Medical University, Wenzhou, Zhejiang, China; ^d^ Pediatric Department, the Second Affiliated Hospital and Yuying Childrens Hospital of Wenzhou Medical University, Wenzhou, Zhejiang, China

**Keywords:** Computed tomography, Crohn’s disease, infliximab, machine learning, sarcopenia

## Abstract

**Background:**

Crohn’s disease (CD) is a chronic inflammatory bowel disease, with infliximab (IFX) commonly used for treatment. However, no clinically applicable model currently exists to predict the response of patients with CD to IFX therapy. Given the strong association between sarcopenia and IFX treatment outcomes, this study developed computerized tomography radiomics-based machine learning (ML) models, utilizing psoas muscle volume as a proxy for skeletal muscle mass, to predict the response of patients with CD to IFX therapy.

**Methods:**

In this retrospective study, patients with CD from two institutions were recruited between January 2010 and January 2023, following stringent inclusion and exclusion criteria. Regions of interest were delineated using 3D Slicer software, and radiomics features were extracted with the Pyradiomics package in Python. *Z* score standardization and independent sample *t* test were applied to identify optimal predictive features, which were then utilized in seven ML algorithms for training and validation. Model performance was assessed through receiver-operating characteristic curves, precision–recall curves, and calibration curve analyses, evaluating accuracy and clinical applicability. Binary logistic regression was employed to identify predictors of IFX treatment response.

**Results:**

A total of 134 patients were included, divided into a training cohort (*n* = 84) and a validation cohort (*n* = 50). Twenty differential radiomics features were selected for integration into the ML models. All models demonstrated strong predictive performance in the validation cohort, with a mean area under the curve of 0.849. The eXtreme Gradient Boosting algorithm outperformed others, achieving an area under the curve of 0.910.

**Conclusion:**

Psoas computerized tomography radiomics-based ML models effectively predict the response of patients with CD to IFX therapy, with the eXtreme Gradient Boosting model exhibiting superior performance.

## Introduction

Crohn’s disease (CD) is a chronic, relapsing inflammatory bowel disease (IBD) with an unclear etiology, capable of affecting the entire gastrointestinal tract [1,2]. Due to reduced food intake, malabsorption, increased energy expenditure, and potential treatment-related side effects, patients with CD often undergo significant changes in body composition [3,4]. Sarcopenia, characterized by the loss of both muscle mass and function, affects up to one-third of patients with CD [5]. The psoas muscle volume is recognized as a partial indicator of overall muscle mass, thus commonly used for assessing sarcopenia [6].

The rising global incidence of CD is imposing a substantial burden on healthcare systems, primarily driven by the use of drugs such as anti-tumor necrosis factor (anti-TNF) biologics [7]. Infliximab (IFX), the first anti-TNF agent approved by the FDA for CD treatment, is widely employed as a first-line therapy [8]. However, a significant proportion of patients with CD (13–40%) experience primary non-response to IFX, and an additional ∼13% experience secondary loss of response annually [9–11]. Predicting the risk of nonresponse prior to IFX therapy is therefore crucial. The factors leading to loss of response to IFX therapy in patients with CD remain incompletely understood. Older age at diagnosis is associated with reduced IFX responsiveness [12], potentially due to age-related changes in immune system composition and gut microbiota, which can alter disease pathogenesis [13]. Patients with CD exhibiting colon involvement are more likely to fail IFX treatment compared to those with isolated ileitis, as a greater proportion of fecal IFX is lost [14,15]. Patients with non-stricturing and non-penetrating CD are more likely to benefit from IFX compared to those with stenosing or fistulizing disease [16,17]. Additionally, individuals with a longer disease duration typically exhibit poorer responsiveness to IFX than those with a shorter disease course [18,19]. This may be ascribed to alterations in mucosal cytokine profiles [20] and advanced fibrosing organ damage [21]. Factors such as body mass index, smoking status, baseline C-reactive protein (CRP) levels, genetic markers, and drug trough levels may also influence response to treatment [22–24]. Notably, sarcopenia has been identified as a potential contributor to nonresponse; however, its role in predicting the response of patients with CD to IFX therapy remains uncertain [25–27].

Machine learning (ML), a branch of artificial intelligence, can capture complex non-linear relationships within data, enabling more accurate predictions than traditional regression models [28,29]. Radiomics extracts numerous quantitative imaging features that serve as inputs for ML algorithms to create predictive models for clinical outcomes [30–33]. While computerized tomography (CT) radiomics-based ML algorithms have been used to predict clinical outcomes in various conditions, few studies have explored their potential in predicting the response of patients with CD to IFX therapy [34–37].

Thus, this study aimed to develop psoas CT radiomics-based ML models to determine their ability to predict the response of patients with CD to IFX therapy.

## Materials and methods

This study was approved by the Review of Ethics Committee in Clinical Research of the First Affiliated Hospital of Wenzhou Medical University (KY2023-R156) and was conducted in compliance with the Declaration of Helsinki. Exemption from informed consent was obtained from the institutional review board due to the retrospective nature of the study.

### Study design and patients

**Figure 1.**
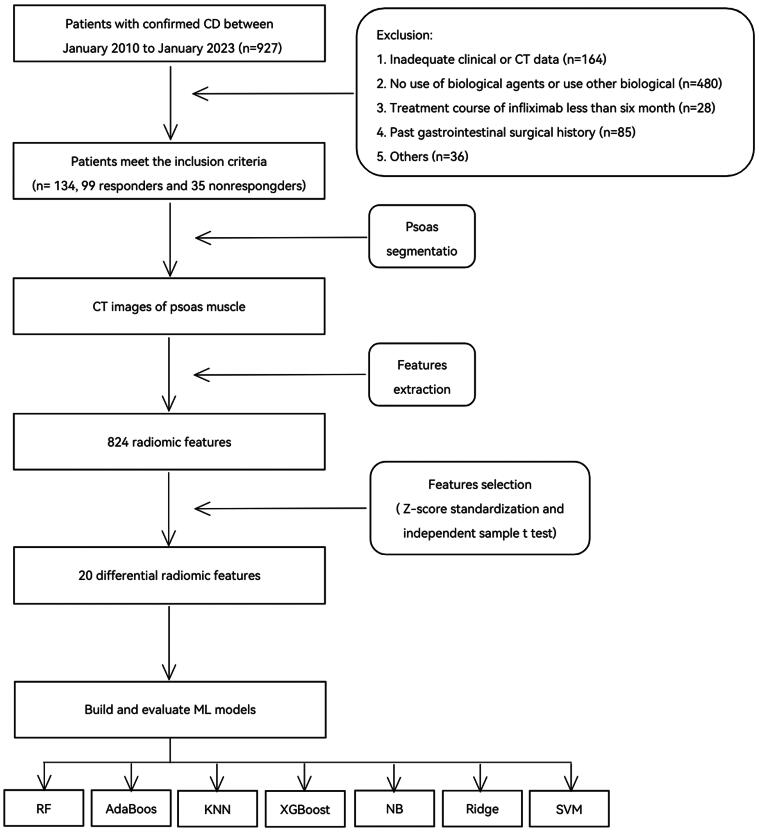
The flowchart of this study.

The flowchart of this study is illustrated in Figure 1. To enhance the study’s credibility and increase the sample size, 134 patients with CD from two medical centers were retrospectively enrolled between January 2010 and January 2023. Patients were categorized into the training and external validation cohorts based on the medical center where they received treatment. Patients from the First Affiliated Hospital of Wenzhou Medical University (Wenzhou, P.R. China) were assigned to the training cohort (*n* = 84), while those from the Second Affiliated Hospital and Yuying Children’s Hospital of Wenzhou Medical University (Wenzhou, P.R. China) were included in the external validation cohort (*n* = 50). Both cohorts adhered to the same inclusion and exclusion criteria. The inclusion criteria were as follows: patients diagnosed with CD based on standard endoscopic, radiographic, and histologic criteria, who received intravenous IFX 5 mg/kg at weeks 0, 2, 6, and every 8 weeks thereafter. The exclusion criteria included the following: absence of CT evaluations performed within 3 months before or after initial IFX treatment, prior treatment with other biologic agents, IFX use for less than 6 months, prior intestinal surgery, incomplete clinical data, severe organ insufficiency, and cancer. CD diagnosis adhered to the European Crohn’s and Colitis Organization guidelines [38].

Demographic data, including age, gender, underlying diseases, smoking and drinking history, and surgical history, were reviewed. Clinical information such as medical history, clinical symptoms, treatment measures, medication regimen, laboratory parameters, imaging data, and endoscopic findings were also collected from electronic medical records.

### Definition of outcomes

Primary nonresponse was defined as the absence of response by week 14, while secondary loss of response was defined as the loss of response after an initial positive response within 52 weeks [11]. Prior to 2017, the conventional treatment course for IFX in both hospitals was 30 weeks, so clinical events were assessed at the 6-month mark in this study. Therapeutic outcomes were categorized as responders (including remission) and nonresponders. Disease activity and response to IFX were evaluated by experienced gastroenterologists using the Crohn’s Disease Activity Index (CDAI) and the Simple Endoscopic Score for Crohn’s Disease (SES-CD) [39]. The SES-CD score was used initially, and if comparable SES-CD scores were unavailable, the CDAI score was applied. Additionally, patients with consistently elevated CRP levels above normal, those requiring CD-related surgery, or those who developed CD-related complications during IFX therapy were classified as nonresponders.

### Psoas segmentation

**Figure 2.**
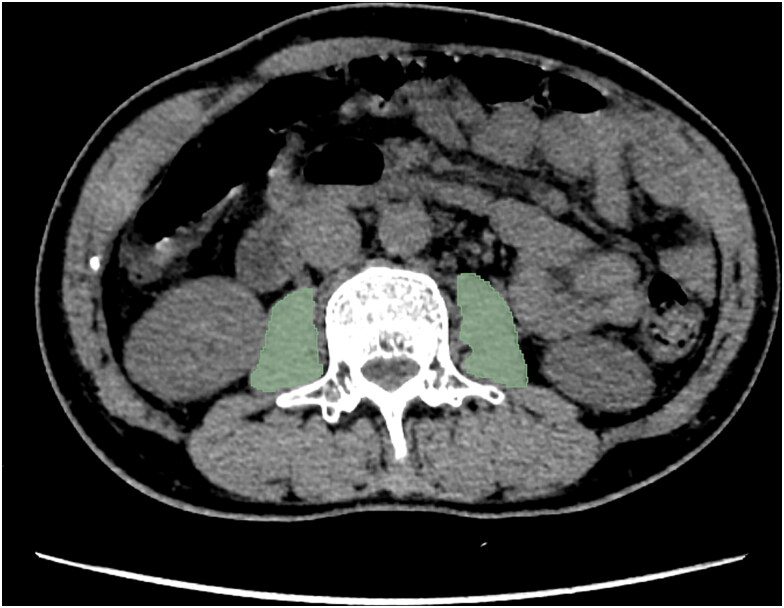
The psoas muscle at the third lumbar level was segmented.

All abdominal CT images were obtained using a dedicated CT scanner (Siemens Healthcare, Forchheim, Germany) and were retrieved from the Picture Archiving and Communication System in DICOM format. The psoas muscle cross-sectional area at the level of the third lumbar vertebrae (L3) is considered an accessible and reliable indicator for assessing sarcopenia [40,41]. Thus, the region of interest (ROI) was manually segmented using the open-source software 3D-Slicer (version 5.0.3) at the L3 level. The segmentation was performed by a specialist with 5 years of experience (Figure 2), and the results were verified by another specialist with seven years of experience one week later. Both specialists were proficient with 3D-Slicer software and were blinded to the study results. Any discrepancies between specialists were resolved through consultation.

### Feature extraction and selection

Radiomics features were extracted from the ROI using Python (version 3.10) with the ‘Pyradiomics’ package, including first-order statistics, shape features, texture features, wavelet-based features, and Laplacian of Gaussian-filtered image features. These extracted features were then normalized using the *Z* score standardization.

The radiomics features were compared between responders and nonresponders using the independent *t* test. Similar variables were removed, and distinct features were retained. The training cohort was used to identify useful features and develop ML models, including random forests (RF), Adaptive Boosting (AdaBoost), K-nearest neighbors (KNN), eXtreme Gradient Boosting (XGBoost), naïve Bayes (NB), Ridge regression (Ridge), and support vector machines (SVM). The external validation cohort was employed to independently evaluate the predictive performance of the models. Receiver-operating characteristic and precision–recall curve analyses were conducted to assess the accuracy of the ML models in both cohorts. Model calibration was evaluated using the Hosmer–Lemeshow goodness-of-fit test.

### Statistical analyses

Results were expressed as mean ± standard deviation or median (range), depending on the data distribution. Continuous data were analyzed using the *t* test or Mann–Whitney *U* test, while categorical data were presented as frequency (percentages) and compared using the chi-square or Fisher’s exact tests. Binary logistic regression was conducted to identify predictors associated with IFX response in patients with CD. A two-sided *p* value of < 0.05 was considered statistically significant for all analyses. Statistical analyses were performed using SPSS 25 (SPSSInc, Chicago, USA), R (version 4.0.3; R Foundation), and Python (version 3.10).

## Results

### Patient features

A total of 134 patients, comprising 106 men and 28 women, with CD who received IFX treatment were enrolled in this study. The mean age of the patients was 24.3 ± 9.3. Among these, 84 patients were categorized in the training group while 50 comprised the external validation group. The incidence of nonresponse among the patients was 26.1%. Patients’ baseline features are presented in Supplementary Table 1 and most features were not statistically significant.

### Extraction of radiomics features

Radiomics features were extracted from the ROI segmentation derived from CT images, resulting in 824 radiomics features. After *Z* score standardization and independent sample *t* test, 20 variables were selected for further analysis (Supplementary Table 2).

### Clinical factors associated with drug response

The baseline characteristics between responders and nonresponders are summarized in Table 1. Significant differences were observed in sex (*p* = 0.001), weight (*p* = 0.001), disease duration (*p* = 0.026), albumin (*p* = 0.004), creatinine (*p* = 0.006), hemoglobin (*p* = 0.013), platelet count (*p* = 0.013), CRP levels (C-reactive protein) (*p* = 0.017), and the CDAI (*p* = 0.012). These variables were further analyzed using binary logistic regression analysis, revealing that high CRP levels were the only significant risk factor for drug response (Supplementary Table 3). The area under the curve (AUC) of CRP for predicting the drug response was 0.627 in the training cohort and 0.660 in the validation cohort (Supplementary Figure 1).

**Table 1.** Baseline characteristics between responders and nonresponders.

	No. (%)			
Variables	Total (*N* = 134)	Responders (*n* = 99)	Non-responders (*n* = 35)	P value
Gender, *n* (%)				0.001
Male	106 (79.10%)	85 (85.86%)	21 (60.00%)	
Female	28 (20.90%)	14 (14.14%)	14 (40.00%)	
Age, years, median (IQR)	23 (18–28)	23 (18–28)	23 (19–28)	0.676
weight, median (IQR)	52.00 (47–58.63)	54.00 (49.00–61.00)	50.00 (43.00–55.00)	0.001
BMI, kg/m^2^, median (IQR)	18.35 (16.73–20.76)	18.71 (16.80–20.96)	17.36 (16.18–19.77)	0.055
Disease duration, months, median (IQR)	9.00 (3.00–24.00)	6.00 (2.00–24.00)	12.00 (8.00–36.00)	0.026
TP, g/L, mean ± SD	68.65 ± 7.82	68.39 ± 7.94	69.35 ± 7.53	0.474
ALB, g/L, median (IQR)	37.95 (33.45–41.53)	39.00 (34.20–42.00)	35.60 (32.00–38.80)	0.004
ALT, U/L, median (IQR)	11.00 (7.75–20.00)	11.00 (8.00–20.00)	9.00 (7.00–15.00)	0.110
AST, U/L, median (IQR)	15.00 (12.00–21.25)	15.00 (13.00–22.00)	15.00 (11.00–18.00)	0.130
Cr, μmol/L, median (IQR)	63.90 (52.35–74.45)	65.50 (56.00–75.40)	54.20 (46.00–71.00)	0.006
BUN, mmol/L, median (IQR)	3.50 (2.90–4.30)	3.50 (3.00–4.50)	3.30 (2.40–4.10)	0.102
Uric acid, μmol/L, mean ± SD	339.77 ± 98.216	349.03 ± 97.54	313.58 ± 96.74	0.066
Neutrophil, 109/L, median (IQR)	5.18 (4.16–6.57)	5.19 (4.18–6.54)	4.93 (3.64–7.18)	0.879
Monocyte, 109/L, median (IQR)	0.56 (0.42–0.80)	0.55 (0.40–0.82)	0.65(0.49–0.78)	0.363
Lymphocyte, 109/L, median (IQR)	1.35 (1.06–1.74)	1.40 (1.10–1.75)	1.24 (1.01–1.67)	0.184
HB, g/L, median (IQR)	128.00 (109.75–136.00)	130.00 (116.00–137.00)	117.00 (103.00–133.00)	0.013
MCV, fl, median (IQR)	83.30 (79.00–86.60)	83.30 (79.20–86.50)	83.00 (78.80–87.40)	0.630
RDW, %, median (IQR)	13.90 (12.90–15.03)	13.90 (12.90–15.00)	14.00 (12.90–15.10)	0.423
Platelet, 109 /L, median (IQR)	327.50 (274.50–422.25)	315.00 (269.00–391.00)	387.00 (311.00–495.00)	0.013
MPV, fl, median (IQR)	9.50 (8.80–10.23)	9.60 (8.90–10.30)	9.30 (8.70–10.00)	0.154
CRP, mg/L, median (IQR)	18.61 (8.31–44.58)	16.10 (7.93–37.50)	34.80 (10.10–64.80)	0.017
ESR, mm/h, median (IQR)	26.50 (14.75–42.00)	25.00 (14.00–40.00)	29.00 (18.00–51.00)	0.214
CDAI, mean ± SD	230.26 ± 118.44	215.11 ± 118.24	273.11 ± 109.62	0.012

IQR, Interquartile range; BMI, Body mass index; TP, Total Protein; SD, standard deviation; ALB, Albumin; ALT, Alanine transaminase; AST, Aspartate Transaminase; Cr, Creatinine; BUN, Urea nitrogen; HB, Hemoglobin; MCV, Mean corpuscular volume; RDW, Red Cell Distribution Width, MPV, Mean platelet volume; CRP, C-reactive protein; ESR, Erythrocyte sedimentation rate; CDAI, Crohn’s Disease Activity Index score.

### Construction of psoas muscle CT radiomics-based machine learning models

**Figure 3.**
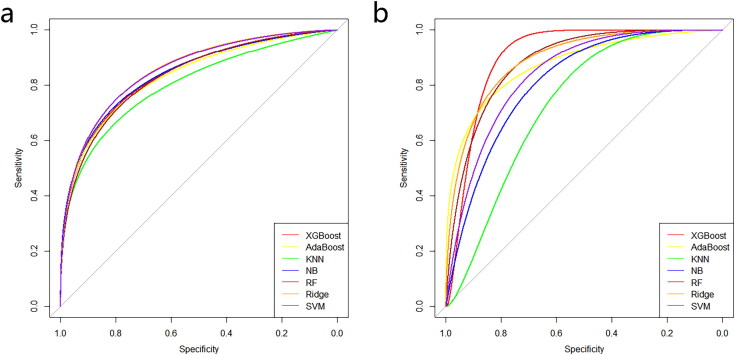
ROC curve analysis of machine learning algorithms for prediction of drug response. (a) Training set. (b) Validation set.

**Figure 4.**
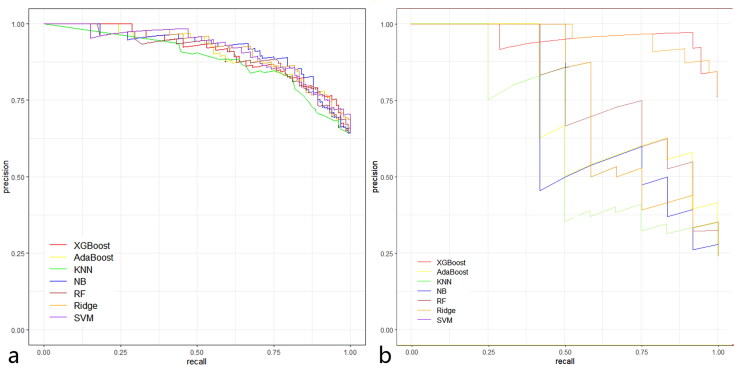
PR curve analysis of machine learning algorithms for prediction of drug response. (a) Training set. (b) Validation set. The *y* axis represents precision and the *x* axis represents recall. If the PR curve of one model is entirely covered by the PR curve of another, the latter model is considered superior, with a higher AP value indicating better the model performance. Abbreviations: PR, precision-recall; AP, area under the precision-recall curve.

Seven ML models, including RF, AdaBoost, KNN, XGBoost, NB, Ridge, and SVM, were developed using the 20 selected radiomics features. All ML models performed well in predicting the drug response in both the training and validation cohorts (Figures 3 and 4). Tables 2 and 3 present the performance metrics (AUC, sensitivity, specificity, accuracy) for each model in the training and validation cohorts, respectively. Among these, XGBoost proved to be the most effective, achieving an AUC of 0.910, sensitivity of 0.921, specificity of 0.917, and accuracy of 0.920 in the validation cohort, although the difference was not statistically significant (Supplementary Table 4). Furthermore, XGBoost showed optimal performance, with an area under the precision-recall curve of 0.961 in the validation dataset. The XGBoost model was also well-calibrated, with a Hosmer–Lemeshow c2 static of 5.28 (*p* = 0.727) in the validation cohort. Overall, the comprehensive analysis indicated that XGBoost is the optimal model for predicting IFX response.

**Table 2.** Diagnostic accuracy for the seven machine learning algorithms with the training cohort of CD patients.

	AUC	AP	Se	Spe	Accuracy
XGBoost	0.843	0.909	75.00	79.73	78.26
AdaBoost	0.836	0.907	74.24	81.08	78.74
KNN	0.797	0.884	76.52	74.32	74.88
NB	0.852	0.915	79.55	82.43	81.64
RF	0.836	0.903	75.76	82.43	80.19
Ridge	0.855	0.915	81.82	77.03	78.26
SVM	0.854	0.915	81.82	75.68	77.77

Abbreviations: CD, Crohn’s disease; AUC, area under the receiver characteristic curve; AP, area under the precision-recall curve; Se, sensitivity; Spe, specificity; SVM, support vector machine; AdaBoost, adaptive boosting; XGBoost, eXtreme gradient machine; NB, Naïve bayes; RF, random forest; KNN, K-nearest neighbors; Ridge, ridge regression.

**Table 3.** Diagnostic accuracy for the seven machine learning algorithms with the validation cohort of CD patients.

	AUC	AP	Se	Spe	Accuracy
XGBoost	0.910	0.961	92.11	91.67	92.00
AdaBoost	0.886	0.741	78.95	91.67	88.00
KNN	0.776	0.632	97.37	50.00	62.00
NB	0.820	0.689	84.21	75.00	78.00
RF	0.893	0.788	76.32	91.67	88.00
Ridge	0.803	0.967	89.47	66.67	70.00
SVM	0.855	0.756	97.37	58.33	66.00

Abbreviations: CD, Crohn’s disease; AUC, area under the receiver characteristic curve; AP, area under the precision-recall curve; Se, sensitivity; Spe, specificity; SVM, support vector machine; AdaBoost, adaptive boosting; XGBoost, eXtreme gradient machine; NB, Naïve bayes; RF, random forest; KNN, K-nearest neighbors; Ridge, ridge regression.

## Discussion

Substantial heterogeneity exists in the therapeutic response to costly anti-TNF-α biologics among patients with CD [42,43]. Consequently, identifying a tool that can accurately predict which patients are less likely to respond to these biologics after a short-term treatment is of significant interest. Such a tool could enable healthcare professionals to identify patients who would benefit from switching to alternative therapies, thereby reducing the costs associated with expensive but likely ineffective treatments, while also mitigating risks related to inadequate disease control and unnecessary steroid use.

Radiomics, an emerging high-throughput imaging post-processing technique, is sensitive to abnormalities not visible to the naked eye [44,45]. CT texture analysis, a widely adopted method in radiomics, provides quantitative textural features by examining the distribution of pixel or voxel gray levels in images [46]. ML, a promising technology with vast potential applications, is increasingly being applied in medical image processing [47,48]. Furthermore, radiomics-based ML, a rapidly advancing field, has been extensively explored in the diagnosis, differentiation, and prognostic prediction of various diseases [49–51]. For instance, Wan et al. used CT-based ML radiomics to predict ovarian cancer survival [52], while Chen et al. employed radiomics-based ML to distinguish low-grade astrocytoma from anaplastic astrocytoma [53]. Building on this, novel CT-based ML models were developed in the present study to assess the response of patients with CD to IFX therapy.

In this study, seven ML models (RF, AdaBoost, KNN, XGBoost, NB, Ridge, and SVM) were constructed using psoas muscle data from third lumbar CT scans to predict the response of patients with CD to IFX therapy. The results demonstrated that all seven models performed excellently in predicting IFX response, with XGBoost performing the best, achieving an AUC value of 0.910. Several models have previously been developed to predict IFX response in patients with CD. For example, Chen et al. created a magnetic resonance enterography-based model [54]. Yang et al. developed a visual nomogram incorporating pancreatic texture features and clinical factors [7]. Liu et al. proposed a model that included sarcopenia parameters to predict loss of response [55]. However, our study is the first to construct CT radiomics-based ML models using muscle CT radiomics features to predict IFX response in patients with CD. The findings of this study further underscore the significant relationship between muscle characteristics and IFX therapy response in patients with CD. Additionally, they provide a novel, accessible, and effective approach for predicting the response to IFX therapy. In the future, abdominal CT scans in patients with CD could serve a dual purpose: diagnosing CD and predicting IFX therapy response. This not only optimizes resource use but also improves patient outcomes, highlighting the potential of precision medicine.

Patients diagnosed with IBD often experience malnutrition, and a key factor associated with this condition is sarcopenia [56,57]. Originally considered a consequence of aging, sarcopenia is now understood to result from various factors, including systemic diseases (e.g. inflammation, endocrine diseases, advanced organ failure, and malignancy), physical inactivity, and insufficient intake of energy or protein [58,59]. Sarcopenia is categorized as primary when it arises from the aging process itself, and secondary when it results from other underlying causes [60]. Notably, sarcopenia has been linked to poor prognosis in various diseases, which has led to increased attention on its role in disease progression [61–64].

Recent studies have highlighted that sarcopenia negatively impacts clinical outcomes in patients with CD, such as leading to higher rates of intestinal resection and postoperative complications [65–67]. Given these findings, the effect of sarcopenia on patients with CD, particularly its relationship with anti-TNF-α therapies, was examined. On one hand, anti-TNF therapy has been shown to reverse sarcopenia [68]; on the other hand, sarcopenia has been associated with nonresponse to anti-TNF treatment in patients with IBD [25,26]. Primary nonresponse to anti-TNF therapy may arise from a pharmacokinetic process (involving low drug levels and high antibodies-to-IFX [ATI] levels) or a pharmacodynamic process (where sufficient drug levels exist but ATI levels remain low) [69,70]. However, the mechanisms by which muscle mass influences the response to IFX therapy in patients with IBD remain incompletely understood. A possible explanation is pharmacokinetic failure [11,25]. Sarcopenia may contribute to anti-TNF pharmacokinetic failure through increased clearance or reduced drug uptake, resulting in inadequate drug levels and a lack of response to therapy [25]. Irisin, a myokine secreted by muscle, has been shown to ameliorate experimental colitis and reduce TNF-α levels in the colon, supporting the notion that muscle mass influences anti-TNF pharmacokinetics [71]. Additionally, a prospective study demonstrated a negative correlation between body muscle parameters and adalimumab level variability, further suggesting that muscle plays a role in anti-TNF pharmacokinetics [72]. The neonatal Fc receptor, which protects IgG from catabolism, can be expressed in skeletal muscle [73,74]. As ATI is an IgG antibody, muscle mass may also impact anti-TNF pharmacokinetics by altering ATI levels. Therefore, muscle mass is closely linked to anti-TNF pharmacokinetics, reinforcing the rationale for using psoas CT radiomics-based ML models to predict the response of patients with CD to IFX therapy.

CRP is an acute-phase protein produced and released by the liver in response to inflammation, primarily triggered by interleukin-6, interleukin-1β, and TNF-α [75]. Serum CRP levels are closely associated with disease activity in CD and are used to predict the response to IFX treatment. However, the findings regarding CRP’s predictive value remain controversial. Some studies suggest that lower CRP levels predict nonresponse to IFX, as they indicate a lack of inflammation, thus implying no therapeutic benefit from anti-TNF treatment [75–77]. Others propose that higher CRP levels predict a poorer response, as they are associated with more severe disease or higher IFX clearance [78]. Our results align with the latter perspective, but because the predictive power of CRP for drug response in this study was significantly lower than that of the ML models, it was excluded from subsequent analysis.

The models effectively predicted IFX therapy response at an early stage; however, several limitations must be acknowledged. First, the limited sample size is a significant constraint. Second, the absence of routine testing for IFX levels and anti-IFX antibody levels presents another important limitation. Third, the lack of consistent CT scans at the L3 level due to variability in equipment and operator experience represents a shortcoming. While external validation from another institution was conducted, multicenter studies are still needed to confirm our findings. Lastly, our ML models only included radiomics features and did not incorporate genetic, clinicopathologic, or molecular biomarker data.

## Conclusion

This study developed seven ML models based on psoas muscle imaging from abdominal CT scans to predict the response of patients with CD to IFX therapy. Among these, the XGBoost model demonstrated the best predictive ability and has significant clinical utility in guiding and optimizing individualized treatment strategies for patients with CD. Future plans include expanding the sample size, collecting data from multiple centers, incorporating IFX levels and anti-IFX antibody levels, and developing a more accurate and reliable model that integrates genetic, clinicopathologic, and molecular biomarker data.

## Supplementary Material

Supplemental Material

Ethical approval document.pdf

Supplementary Table 2.docx

Supplementary Table 1.docx

Supplementary Table 4.docx

Supplementary Table 3.docx

## Data Availability

The datasets analyzed in the study are available from the corresponding author (Fujun Yu) on reasonable request.

## Abbreviations

CD: Crohn’s disease
IFX: Infliximab
ML: Machine learning
XGBoost: Extreme gradient boosting
IBD: Inflammatory bowel disease
TNF-α: Tumor necrosis factor-α
CT: Computed tomography
CDAI: Crohn’s Disease Activity Index
SES-CD: Simple Endoscopic Score for Crohn’s Disease
L3: The third lumbar vertebrae
ROI: Region of interest
RF: Random forest
AdaBoost: Adaptive Boosting
KNN: K-nearest neighbors
XGBoost: eXtreme Gradient Boosting
NB: Naïve Bayes
Ridge: Ridge regression
SVM: Support vector machines
CRP: C-reactive protein
AUC: Area under the curve
ATI: Antibody-to-IFX
