# Supplementary material for: Psoas muscle CT radiomics-based machine learning models to predict response to infliximab in patients with Crohn’s disease
Source: Ann Med. 2025 Jul 5;57(1):2527954. doi: 10.1080/07853890.2025.2527954 (PMC12231329; doi:10.1080/07853890.2025.2527954)

**Supplementary Figure 1.** ROC curve analysis of CRP for prediction of drug response (a) Training set. (b) Validation set


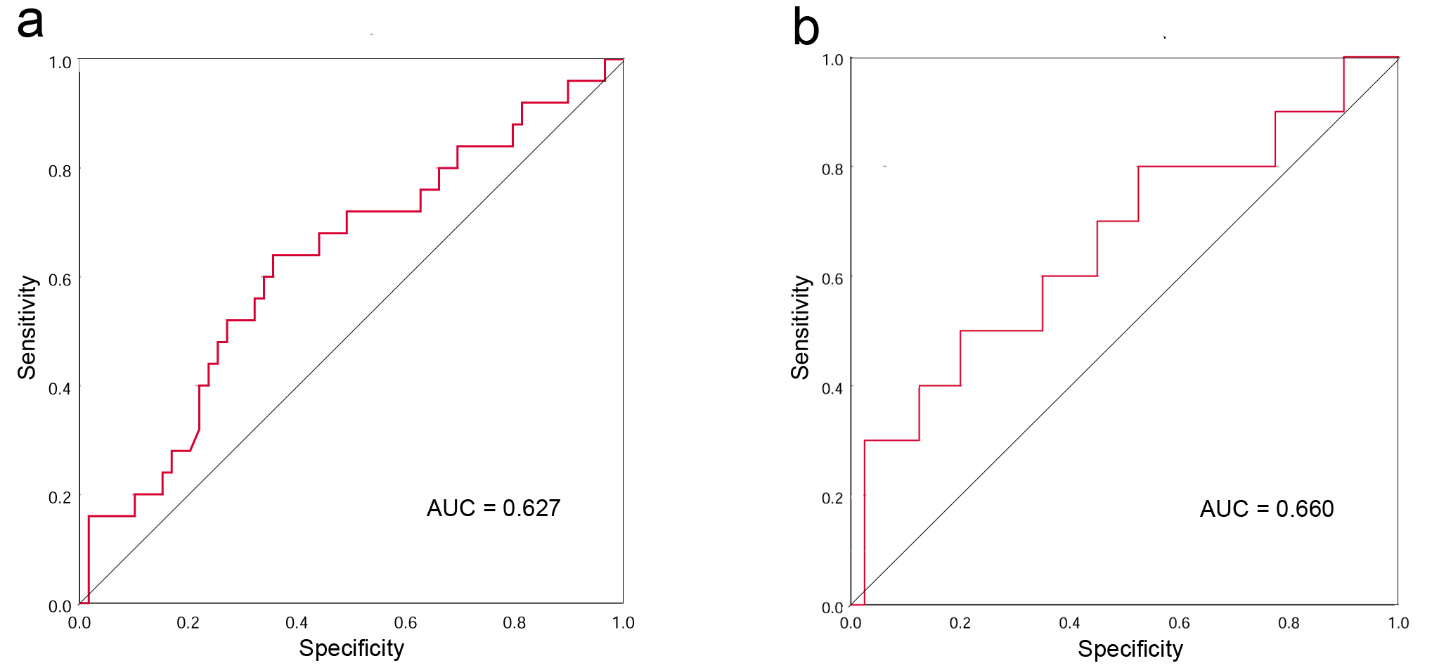

Supplement: Supplemental Material [file IANN_A_2527954_SM4949.docx]
