# Supplementary material for: Psoas muscle CT radiomics-based machine learning models to predict response to infliximab in patients with Crohn’s disease
Source: Ann Med. 2025 Jul 5;57(1):2527954. doi: 10.1080/07853890.2025.2527954 (PMC12231329; doi:10.1080/07853890.2025.2527954)
Supplement: Supplementary Table 2.docx [file IANN_A_2527954_SM3767.docx]

| Features’ name |
| --- |
| original_glcm_Imc1 |
| original_glrlm_GrayLevelNonUniformity |
| wavelet-LLH_gldm_SmallDependenceEmphasis |
| wavelet-LLH_gldm_SmallDependenceLowGrayLevelEmphasis |
| wavelet-LLH_glszm_ZonePercentage |
| wavelet-LHH_glszm_ZonePercentage |
| wavelet-HLH_glrlm_HighGrayLevelRunEmphasis |
| wavelet-HLH_glrlm_LowGrayLevelRunEmphasis |
| wavelet-HLH_glszm_ZoneEntropy |
| wavelet-HHL_gldm_DependenceNonUniformityNormalized |
| wavelet-HHL_gldm_DependenceVariance |
| wavelet-HHH_gldm_LargeDependenceHighGrayLevelEmphasis |
| wavelet-HHH_glrlm_LongRunEmphasis |
| wavelet-HHH_glrlm_LongRunHighGrayLevelEmphasis |
| wavelet-HHH_glszm_HighGrayLevelZoneEmphasis |
| wavelet-HHH_glszm_LowGrayLevelZoneEmphasis |
| wavelet-HHH_glszm_SizeZoneNonUniformity |
| wavelet-HHH_glszm_SizeZoneNonUniformityNormalized |
| wavelet-HHH_glszm_SmallAreaEmphasis |
| wavelet-HHH_glszm_SmallAreaLowGrayLevelEmphasis |

**Supplementary Table 2.** [Twenty](javascript:;) differential radiomics features
