# Supplementary material for: Psoas muscle CT radiomics-based machine learning models to predict response to infliximab in patients with Crohn’s disease
Source: Ann Med. 2025 Jul 5;57(1):2527954. doi: 10.1080/07853890.2025.2527954 (PMC12231329; doi:10.1080/07853890.2025.2527954)
Supplement: Supplementary Table 1.docx [file IANN_A_2527954_SM3766.docx]

**Supplementary Table 1.** Baseline characteristics between training and validation set

|  | | No. (%) | | | | | | |
| --- | --- | --- | --- | --- | --- | --- | --- | --- |
| Variables | | Total (N = 134) | Training set (n=84) | Validation set (n=50) | P value |  |  |  |
| Gender, n (%) | |  |  |  | 0.525 |  |  |  |
|  | Male | 106 (79.10%) | 101 (82.8%) | 29 (60.4%) |  | |  |  |
|  | Female | 28 (20.90%) | 21 (17.2%) | 19 (39.6%) |  | |  |  |
| Age, years, median (IQR) | | 23 (18-28) | 25 (20-29) | 19 (14.75-24) | <0.001 |  |  |  |
| weight, kg, median (IQR) | | 52.00 (47.00-58.625) | 52.00 (47.25-58.00) | 53 (46.75-61.63) | 0.927 |  |  |  |
| BMI, kg/m2, median (IQR) | | 18.35 (16.73-20.76) | 17.98 (16.69-20.62) | 18.85 (16.72-20.81) | 0.422 |  |  |  |
| Disease duration, months, median (IQR) | | 9.00 (3.00-24.00) | 10.00 (4.00-25.50) | 6.00 (2.00-21.25) | 0.065 |  |  |  |
| Current Smokers, n (%) | | 9 (6.72%) | 5 (5.95%) | 4 (8.00%) | 0.184 |  |  |  |
| TP, g/L, mean ± SD | | 68.65±7.82 | 68.89±8.39 | 68.23±6.83 | 0.636 |  |  |  |
| ALB, g/L, mean ± SD | | 37.31±6.37 | 35.94±6.54 | 39.62±5.38 | 0.001 |  |  |  |
| ALT, U/L, median (IQR) | | 11.00 (7.75-20.00) | 11.00 (7.25-20.00) | 10.00 (7.75-18.00) | 0.395 |  |  |  |
| AST, U/L, median (IQR) | | 15.00 (12.00-21.25) | 15.50 (12.00-21.00) | 14.00 (12.00-22.00) | 0.382 |  |  |  |
| Cr, μmol/L, median (IQR) | | 63.90 (52.35-74.45) | 67.00 (55.00-76.75) | 61.15 (48.43-70.90) | 0.036 |  |  |  |
| BUN, mmol/L, median (IQR) | | 3.50 (2.90-4.30) | 3.50 (2.90-4.30) | 3.50 (2.88-4.25) | 0.814 |  |  |  |
| Uricacid, μmol/L, mean ± SD | | 339.77±98.22 | 332.59±102.78 | 351.85 ±89.73 | 0.274 |  |  |  |
| Neutrophil, 109 /L, median (IQR) | | 5.18 (4.16-6.57) | 5.15 (4.05-6.43) | 5.31 (4.27-6.84) | 0.590 |  |  |  |
| HB, g/L, median (IQR) | | 128.00 (109.75-136.00) | 124.50 (109.00-135.00) | 131.50 (113.00-138.00) | 0.292 |  |  |  |
| MCV, fl, median (IQR) | | 83.30 (79.00-86.60) | 83.25 (79.05-86.68) | 83.65 (78.90-86.53) | 0.740 |  |  |  |
| RDW, %, median (IQR) | | 13.90 (12.90-15.03) | 14.10 (13.10-15.10) | 13.35 (12.68-14.80) | 0.068 |  |  |  |
| Platelet, 109 /L, median (IQR) | | 327.50 (274.50-422.25) | 340.50 (280.75-427.50) | 313.50 (256.75-398.75 ) | 0.344 |  |  |  |
| MPV, fl, median (IQR) | | 9.50 (8.80-10.23) | 9.55 (8.83-10.30) | 9.20 (8.50-10.10) | 0.230 |  |  |  |
| CRP, mg/L, median (IQR) | | 18.61 (8.31-44.58) | 19.10 (8.46-43.75) | 18.20 (8.00-50.68) | 0.954 |  |  |  |
| ESR, mm/h, median (IQR) | | 26.50 (14.75-42.00) | 24.50 (12.75-39.51) | 30.50 (15.50-44.25) | 0.240 |  |  |  |
| CDAI, mean ± SD | | 230.26±118.44 | 260.92±110.77 | 178.75±113.89 | <0.001 |  |  |  |
| Nonresponse, n (%) | | 35 (26.12%) | 25 (29.76%) | 10 (20.00%) | 0.213 | | |  |

Abbreviations: IQR, Interquartile range; BMI, Body mass index; TP, Total Protein; SD, standard deviation; ALB, Albumin; ALT, Alanine transaminase; AST, Aspartate Transaminase; Cr, Creatinine; BUN, Urea nitrogen; HB, Hemoglobin; MCV, Mean corpuscular volume; RDW, Red Cell Distribution Width, MPV, Mean platelet volume; CRP, C-reactive protein; ESR, Erythrocyte sedimentation rate; CDAI, Crohn’s Disease Activity Index score.
