# Supplementary material for: Psoas muscle CT radiomics-based machine learning models to predict response to infliximab in patients with Crohn’s disease
Source: Ann Med. 2025 Jul 5;57(1):2527954. doi: 10.1080/07853890.2025.2527954 (PMC12231329; doi:10.1080/07853890.2025.2527954)
Supplement: Supplementary Table 4.docx [file IANN_A_2527954_SM3765.docx]

| **Supplementary Table 4. Statistical differences between XGBoost and other ML models in predicting the response of CD patients to IFX treatment in the training and validation cohorts** | | | |
| --- | --- | --- | --- |
|  | P values |  |  |
| Cohorts | Training cohort | Validation cohort |  |
| XGBoost |  |  |  |
| AdaBoost | 0.6324 | 0.5643 |  |
| KNN | 0.0998 | 0.1197 |  |
| NB | 0.6151 | 0.0544 |  |
| RF | 0.6909 | 0.5411 |  |
| Ridge | 0.1620 | 0.8710 |  |
| SVM | 0.4983 | 0.2933 |  |

Abbreviations: XGBoost, eXtreme gradient machine; ML, machine learning; CD, Crohn’s disease; IFX, infliximab; AdaBoost, adaptive boosting; KNN, K-nearest neighbors; NB, Naïve bayes; RF, random forest; Ridge, ridge regression; SVM, support vector machine.

|  |
| --- |
