# Supplementary material for: Psoas muscle CT radiomics-based machine learning models to predict response to infliximab in patients with Crohn’s disease
Source: Ann Med. 2025 Jul 5;57(1):2527954. doi: 10.1080/07853890.2025.2527954 (PMC12231329; doi:10.1080/07853890.2025.2527954)
Supplement: Supplementary Table 3.docx [file IANN_A_2527954_SM3763.docx]

**Supplementary Table 3.** Outputs of the binary logistic regression for potential predictors associated with drug response

| Variables | B | SE | Wald | df | p | Odds Ratio | 95% CI of Odds Ratio | |
| --- | --- | --- | --- | --- | --- | --- | --- | --- |
|  |  |  |  |  |  |  | Lower | Upper |
| sex | -0.857 | 0.641 | 1.785 | 1 | 0.182 | 0.424 | 0.121 | 1.492 |
| weight | 0.012 | 0.007 | 2.721 | 1 | 0.099 | 1.012 | 0.998 | 1.027 |
| disease duration | -0.050 | 0.031 | 2.710 | 1 | 0.100 | 0.951 | 0.896 | 1.010 |
| ALB | -0.031 | 0.053 | 0.342 | 1 | 0.558 | 0.970 | 0.874 | 1.075 |
| Cr | -0.010 | 0.019 | 0.291 | 1 | 0.589 | 0.990 | 0.954 | 1.027 |
| HB | 0.011 | 0.018 | 0.387 | 1 | 0.534 | 1.011 | 0.976 | 1.048 |
| platelet | 0.002 | 0.002 | 0.922 | 1 | 0.337 | 1.002 | 0.998 | 1.006 |
| CRP | 0.016 | 0.008 | 4.413 | 1 | 0.036 | 1.016 | 1.001 | 1.032 |
| CDAI | -.001 | 0.003 | 0.055 | 1 | 0.815 | 0.999 | 0.994 | 1.005 |

Abbreviations: ALB, Albumin; Cr, Creatinine; HB, Hemoglobin; CRP, C-reactive protein; CDAI, Crohn’s Disease Activity Index score.
